# Universal medical image segmentation via in-context cross-attention

**DOI:** 10.3389/frai.2025.1698324

**Published:** 2025-11-25

**Authors:** Costin F. Ciusdel, Alex Serban, Tiziano Passerini

**Affiliations:** 1 Foundational Technologies, Siemens SRL, Brasov, Romania; 2 Siemens Healthineers, Princeton, NJ, United States

**Keywords:** universal semantic segmentation, in-context cross attention, medical imaging, deep learning, neural networks

## Abstract

Semantic segmentation is critical in medical image processing, with traditional specialist models facing adaptation challenges to new tasks or distribution shifts. While both generalist pre-trained models and universal segmentation approaches have emerged as solutions, universal methods offer advantages in versatility, sample efficiency, and integration ease into annotation pipelines. We introduce a novel universal segmentation method based on the premise that pre-selecting relevant regions from support sets improves segmentation accuracy. Our approach implements cross-attention between query images and support set images, coupled with an innovative attention up-scaling mechanism that efficiently computes cross-attention on small-scale features with upscaling to higher resolutions. The design inherently supports explainability by allowing inspection of relevant support set locations for each input region. Extensive evaluation across 29 medical datasets spanning 9 imaging modalities and 135 segmentation tasks demonstrates consistent performance improvements, even with lightweight models. Our experiments show proportional gains in segmentation performance as support set size increases, with the cross-attention mechanism effectively selecting the most relevant support images from larger annotation pools. Additionally, our explainability module demonstrates competitive or improved interpretability when compared to established methods like LayerCAM.

## 1 Introduction

Semantic segmentation plays an important role in medical image processing, and involves the classification of pixels into meaningful groups based on their semantic relationships. Conventional segmentation methods, often referred to as *specialist* models (Azad et al., 2024; Zhang et al., 2023), are designed to execute a single segmentation task and are trained using labeled data. Although these methods achieve satisfactory performance for a multitude of tasks (Azad et al., 2024; Srinivasan et al., 2024; Liu X. et al., 2024; Huang L. et al., 2024; Isensee et al., 2024), adapting them to new tasks or distribution shifts requires the development of new models and the acquisition of new training data that matches the new distributions. This process is known to be both costly and time-consuming (Galbusera and Cina, 2024).

To address these challenges, the development of larger-scale, *generalist* models through pre-training (Tu et al., 2024; Zhu J. et al., 2024; Zhang and Metaxas, 2024), or of *universal* segmentation models (Butoi et al., 2023; Takaya and Yamamoto, 2024), emerged as promising solutions. Pre-trained models leverage pretext tasks such as masked-image-modeling (Chen et al., 2023), image-text contrastive learning (Lin et al., 2024), or prompt-based segmentation (Ma et al., 2024) to build models that can be robustly applied across existing and novel tasks. Within this category, some models require fine-tuning for new tasks, such as those using masked-image-modeling or image-text pre-training, while others require user interaction to define new sets of pixels for segmentation e.g., through bounding boxes or text prompts (Ma et al., 2024; Wang et al., 2023).

In contrast, universal models require only a few annotated samples, which are provided as examples. The models learn to use these examples to segment new images in a single forward pass. From this perspective, the initial annotations can be acquired using an interactive model, such as a pre-trained prompt-based model (Ma et al., 2024). In this article, we focus on the development of novel universal segmentation methods, as their pre-training requires less samples than generalist models (Ma et al., 2024). Moreover, universal methods are generally more versatile, require less user interactions, can be recursively used to improve performance as more data is annotated by increasing the support set size, are lighter, and can easily be integrated in active learning annotations pipelines.

We introduce a novel method based on the assumption that pre-selecting relevant regions of interest from the support set leads to improved segmentation masks, as not all parts of the input are relevant for the task at hand. To achieve this, we introduce a cross-attention mechanism between the query image and the support set images, and a novel attention up-scaling mechanism. This mechanism enables efficient use of cross-attention in encoder-decoder architectures, enabling us to compute cross-attention on small-scale features and upscale it to higher resolutions. We provide detailed reasoning behind our architectural choices and demonstrate how these decisions inherently support explainability, allowing us to inspect the relevant support set locations for each input region.

We evaluate our method on an extensive collection of medical datasets, comprising 29 datasets across 9 imaging modalities, resulting in a total of 135 segmentation tasks. Our approach demonstrates consistent performance improvements across the majority of tasks, even when using a lightweight model. Additionally, we investigate the impact of increasing the number of support set images and observe proportional gains in segmentation performance. The cross-attention mechanism further enables the selection of the most relevant support images from a larger pool of annotated data, improving overall accuracy. We also assess the effectiveness of our explainability module by comparing it against established methods such as LayerCAM (Jiang et al., 2021), showing competitive and improved interpretability.

The remainder of this article is organized as follows. We discuss background and related work (Section 2) followed by a description of the method and architecture, data, and training details (Section 3). Next, we introduce qualitative and quantitative evaluations of the model (Section 4) and conclude with a discussion (Section 5).

## 2 Related work

As discussed in Section 1, we identify two primary categories of methods focused on developing generalist or universal segmentation models. Within the first category, notable examples include works based on the *medical segment anything* model (Ma et al., 2024; Zhu J. et al., 2024; Zhou et al., 2024). This model is pre-trained to segment over 1.5 million medical images across 10 different modalities, encompassing a wide range of abnormalities, including cancer (Ma et al., 2024). Such models typically use a large backbone and a lightweight decoder, and incorporate a prompt encoder that uses inputs such as a bounding box or specific points from the object to be segmented. By training on a diverse dataset, the model acquires the ability to generalize across various medical imaging scenarios, demonstrating robust performance in segmenting different types of medical images with or without prompts. (Wu and Xu, 2024) build upon this method by using a segmented image as a prompt, further improving the model's capability to adapt to specific segmentation tasks based on visual cues.

Within the second category, we find related works focusing on conditioned and in-context medical image segmentation (Butoi et al., 2023; Zhu Y. et al., 2024; Rakic et al., 2024; Yuan and Cheng, 2024). Notably, (Butoi et al., 2023) were among the first to propose a universal segmentation model for medical images, closely aligning with our approach. Their architecture employs a cross-convolutional block that facilitates information transfer between the annotated set of images (also known as the support set) and the image to be annotated (query). This block is integrated into an encoder-decoder architecture with skip connections, similar to a U-Net model. The model is trained on a dataset comprising over 50 independent datasets, spanning more than 26 domains and 16 modalities, demonstrating robust performance across a wide range of tasks. The authors also illustrate that increasing the size of the support set enhances performance, indicating that the model's effectiveness improves with more annotated examples. Rakic et al. (2024) further develop this method by generating a mean representation from the support set and applying the cross-convolutional block twice: first between the query and the mean candidate, and then between the candidates and their mean representation. While this procedure improve segmentation performance for some tasks, it does not consistently improve results across all tasks.

Other articles use partitioning or clustering algorithms to perform few shot segmentation. For example, Zhu Y. et al. (2024) use prototype learning to partition the input image pixels according to the support set masks, group and classify the pixels into desired labels. Huang W. et al. (2024) present a similar technique where the prototypes are optimized using graph reasoning. Liang et al. (2023) define pixel clusters based on the query matrix of a transformer architecture and iteratively optimize the clusters using expectation maximization in order to attribute each pixel to a cluster corresponding to a label. Niu et al. (2024) develop a more comprehensive framework for unsupervised image segmentation where they generate pseudo-lables from pre-trained self-supervised models followed by clustering difference pixel-based semantics.

Wong et al. (2024) introduce a unified approach that builds upon the work of Wu and Xu (2024), leveraging multiple cues for segmentation. Specifically, the segmentation process begins with point or box-based prompts, similar to the segment anything model (Ma et al., 2024). As annotations are collected, both the images and new segmentations are incorporated as inputs, offering more detailed context and visual cues. To facilitate this, the authors improve the architecture proposed by Butoi et al. (2023) to accommodate additional cue types, such as boxes or clicks. They demonstrate that increasing the number of images annotated decreases the need to provide additional prompts or cues. Du et al. (2024) use both visual and textual cues, but project and process them together with the image features through a transformer backbone which outputs segmentation mask embeddings. This allows the model to process diverse cues using the attention mechanism, and select the most important ones for the current task. Liu Y. et al. (2024) use a bidirectional pre-fusion of features between visual and language queues, before passing them through a multi-modal transformer and a segmentation decoder that outputs the final segmentation mask. Compared with Du et al. (2024), this approach disentangles the features from images and text, allowing features from language and vision to be processed by different encoders.

Cheng et al. (2025) propose an attention mechanism applied across different blocks of a U-Net-like CNN architecture to dynamically select the most relevant features within each block. This design enables a lightweight alternative to transformer-based models by relying solely on convolutional operations. In contrast, our cross-attention mechanism is even more efficient, as it is applied only once at the final stage of the encoder, significantly reducing computational overhead while still allowing effective feature selection from support examples.

A related concept is found in Mask2Former (Cheng et al., 2022), where pixel-level features extracted from a vision encoder are combined via a decoder-only transformer. In this case, learned queries interact with the pixel embeddings through cross-attention to generate mask predictions. However, Mask2Former relies on a full transformer decoder with multiple layers and attention heads, leading to increased model complexity.

## 3 Methods

This section outlines the model architecture, describes the datasets used for training and evaluation, and details the training configuration employed in our experiments.

### 3.1 Model architecture

Our method is based on the hypothesis that not all regions within the support set images are relevant when segmenting a specific region inside the query image. Additionally, the relevant regions from the support set may occupy different spatial positions. For example, segmenting a query organ located on the left side of the query image might necessitate information from the right side of the support images (e.g., due to panning motions during the acquisition). To address this, we propose a mechanism that selects the most relevant support set locations for each query image region, irrespective of their spatial proximity. This allows the model to adaptively focus on the most informative areas for each task.

This contrasts with previous models (e.g., Butoi et al., 2023; Rakic et al., 2024), which do not include any feature selection mechanisms and instead rely solely on convolutional layers. As a result, they are constrained by limited local receptive fields and require deep network stacks to achieve a sufficiently large field of view, making it challenging to capture large spatial offsets between query regions and relevant support regions.

To implement this mechanism, we use attention maps, as they are both flexible (i.e., changing the support set sizes does not require retraining), act globally (i.e., they can attend to all support set locations regardless of their spatial position) and have the capacity to select relevant regions (i.e., the attention values are used to weight the relevant regions and attenuate uninformative regions). Nonetheless, since segmentation models typically use an encoder-decoder architecture with skip connections, attention maps need to be computed across multiple feature map scales. Directly computing large attention maps, however, is complex and computationally demanding. To mitigate this challenge, we introduce an up-sampling mechanism that enables attention maps from lower-dimensional layers to be reused in higher-dimensional layers.

**Figure 1:**
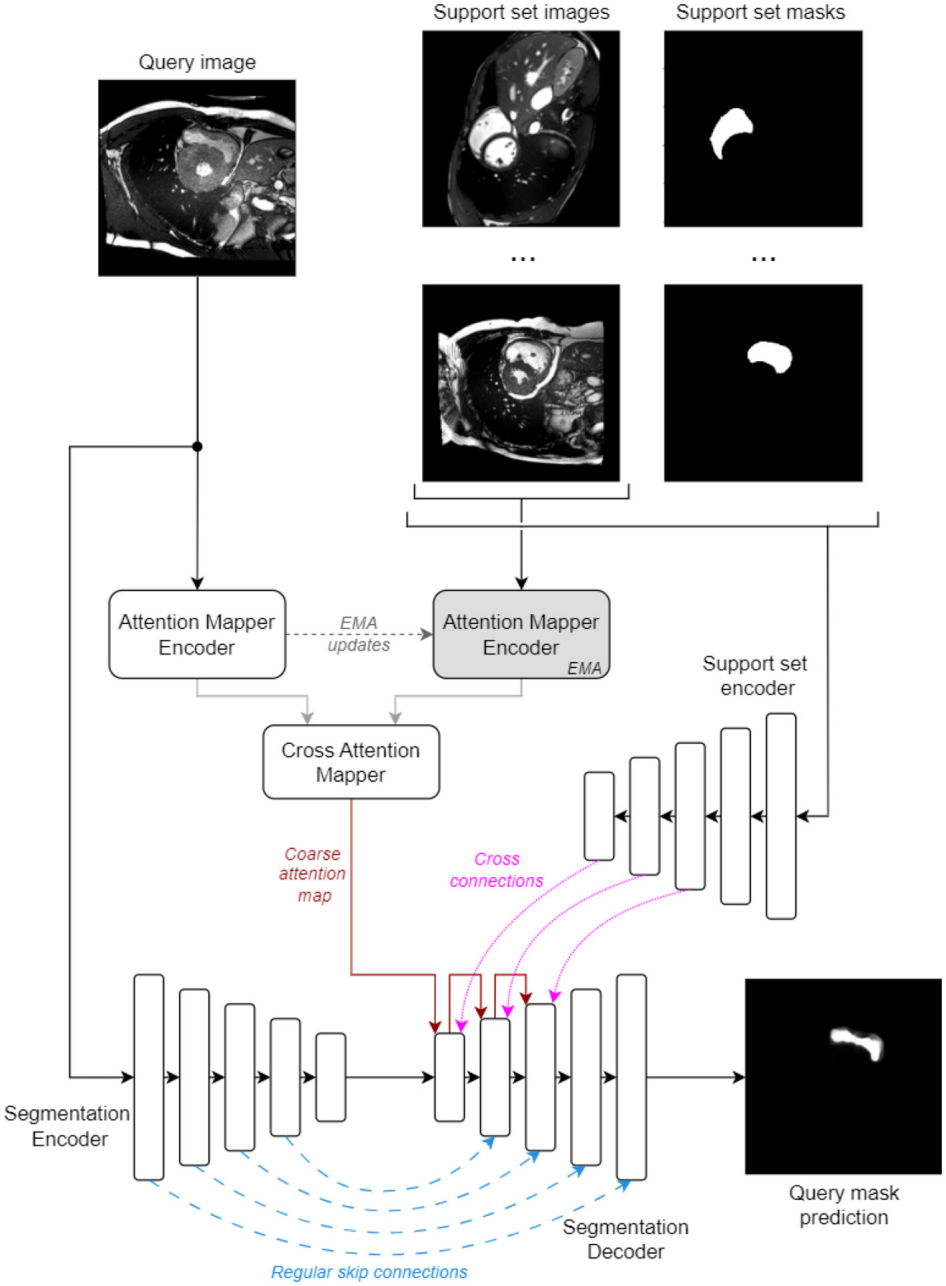
Proposed model's overall architecture.

An overview of the proposed architecture is illustrated in Figure 1. Briefly, it has an encoder-decoder structure with regular skip connections (from early layers of the *Segmentation Encoder*) and cross skip connections (from the *Support Set Encoder*). The attention maps are applied in the *Segmentation Decoder* using the up-sampling mechanism to modulate the cross skip connections and weight the relevance of the features from all spatial support locations.

These maps must reflect the semantic similarity between the regions in the query image and the important regions in the support set images that will contribute to the generated mask, and can act as an explainability tool for understanding which regions contribute to the final predictions. We enforce this behavior by introducing a series of constraints into the proposed model architecture: (i) information from the support set, which includes the segmentation masks, is encoded by an independent *Support Set Encoder* and only decoded using the attention map modulation; (ii) the attention maps are built as an all-to-all mapping (where all query regions can potentially attend all support set regions) in a task-agnostic manner and rely only on visual similarity between the query and support set images. We use independent encoders for the attention map encoders and the segmentation encoder to avoid any sub-model cross-talk outside the modulation done by the attention maps.

The model processes images resized to 256 × 256 pixels and can use support sets containing a variable number *S* of task-specific image and mask pairs.

#### 3.1.1 Coarse attention map

The attention map is computed as follows: a MSCAN (Guo et al., 2022) encoder (*Attention Mapper Encoder*, Figure 1) is applied on the query image to produce a feature pyramid with output-strides of 8, 16, and 32 (sizes of 32 × 32, 16 × 16, and 8 × 8). Concurrently, an EMA (EMA) version of this encoder processes all support set images to produce a pyramid of the same dimensions, now incorporating features from all *S* support images. The coarsest feature map from both pyramids are fed into a *Cross Attention Mapper* block. This block operates similarly to a one-head transformer attention stage. It computes a coarse attention map *A* of shape (*H*×*W, S*×*H*×*W*), where *H* and *W* are the spatial sizes of the coarse feature maps (i.e., 8 × 8), as follows:


$$\begin{array}{l} A_{i}=\sigma \left(\frac{X_{i}^{q}\cdot W_{Q}\cdot W_{K}^{T}\cdot {X^{s}}^{T}}{\delta \cdot T}\right) \end{array}$$ (1)

where σ is the softmax operator, δ is a scaling parameter equal to 0.1*d* (*d* is the number of feature channels), *T* is a learnable softmax temperature, $X_{i}^{q}$ is the i-th row of the query embedding *X*^*q*^∈ℝ^*HW*×*d*^, *X*^*s*^∈ℝ^*SHW*×*d*^ is the support embedding and $W_{Q},W_{K}\in {\mathbb{R}}^{d\times d}$ are trainable parameters. The i-th row of *X*^*q*^ is the embedding of location *i* in the flattened *H*×*W* tensor of the coarse query feature map.

In this formulation, the row-wise sum of *A* is 1, ensuring that every location in the query image receives a weighted sum of features from all locations in the support set, with the weights totaling 1. This approach forces a query location (i.e., a specific region in the query image) that lacks adequate representation in the support set (i.e., no similar regions exist within the support images) to still attend to the support set, resulting in a weighted combination of potentially irrelevant support features.

To mitigate such scenarios, we use a mechanism similar to registry tokens (Darcet et al., 2024), and modify Equation 1 to allow each row *A*_*i*_ to sum to a value less than or equal to 1 by introducing a series of learnable synthetic tokens, which are concatenated along the row dimension of *X*^*s*^. This allows a query location to attend to one or more of these tokens when the support set lacks relevant samples, reducing the support set's influence on that specific query location. The revised attention formula is now:


$$\begin{array}{l} A_{i}=\sigma \left(\frac{X_{i}^{q}\cdot W_{Q}\cdot W_{K}^{T}\cdot {\left[X^{s},t^{s}\right]}^{T}}{\delta \cdot T}\right)\left[:S\times H\times W\right] \end{array}$$ (2)

where [*X*^*s*^, *t*^*s*^] means concatenation of the *t*^*s*^ tokens along the row dimension of *X*^*s*^ and [:*S*×*H*×*W*] signifies keeping only the first *S*×*H*×*W* entries (i.e., discarding the attention weights for the *t*^*s*^ tokens).

The use of EMA version of *Attention Mapper Encoder* for computing *X*^*s*^ prevents *A*_*i*_ from collapsing into a single fixed vector, ∀*i*∈{1..*SHW*} (the case where all query locations attend to the same support location, regardless of their semantic meaning). Moreover, the encoder processes only images (disregarding the target masks from the support set), so the attention mapping is based only on the visual semantic similarity between the query and support image regions. Once the attention map *A* is computed for a specific tuple of (query image, support images), changing the target masks (i.e., segmenting another organ in the same images) does not require re-computing *A*.

#### 3.1.2 Feature encoding and segmentation decoding

As discussed above, to embed the support set images and their corresponding masks, we use an independent *Support Set Encoder*. This encoder takes the concatenated support images and their corresponding target masks as input. The concatenation is done along the channel dimension because the images and masks are spatially correlated, meaning they have a pixel-to-pixel correspondence. The input tensor has the shape *S*, 2, *H*_*img*_, *W*_*img*_, where the two channels result from the concatenation, and *H*_*img*_ = *W*_*img*_ = 256 represent the pixel dimensions. All support set examples are encoded into feature pyramids with the same dimensions as those computed by the attention encoders (Section 3.1.1).

To extract segmentation relevant features, the query image is processed by the independent *Segmentation Encoder*. This encoder shares the same architecture as the *Support Set Encoder*, except for the number of input channels, as it processes only images. Additionally, their architecture was devised to obtain a smaller FoV (FoV) at each stage by simplifying the original MSCAN (Guo et al., 2022) blueprint. For this, (i) we dropped the usage of all multi-scale convolutional attention layers (Guo et al., 2022), and (ii) set the kernel size for all feed-forward networks (FFN) to 1 instead of 3, except for the first FFN in each encoder stage. The resulting FoV sizes (as ratios of the input image size) inside the feature pyramids produced by the support set and segmentation encoders are as follows: 2.7% after the stem stage (output stride 2), 7.4% after stage 1 (output stride 4), 16.8% after stage 2 (output stride 8), 35.5% after stage 3 (output stride 16) and 73% after stage 4 (output stride 32).

The smaller FoV aims to localize attention maps, ensuring that features from a support set location are highly representative of its immediate vicinity. This localization is important for model explainability, when inspecting attention maps at various output-stride levels in the segmentation decoder. A larger FoV in the support set and segmentation encoders could capture unwanted long-range dependencies, diluting the attention maps' effectiveness. By maintaining a small FoV, the attention maps remain highly relevant and descriptive for each query and support location. However, studies show that a large FoV can enhance segmentation performance by providing more context around each pixel (Chen et al., 2017; Nekrasov et al., 2018). To compensate for the encoders' small FoV and achieve low training losses, the model relies on the cross-attention maps, which offer a global FoV.

To integrate the projected features and compute the final segmentation map, we employ a single *Segmentation Decoder*. This module consists of five stages: the first three stages integrate the pre-computed coarse cross-attention map with the segmentation and support set features, while the final two stages are standard up-scaling stages that rely solely on query skip connections. In the first three stages, interaction with the support set is enabled through the *Provided-Attention Blocks*, as follows:


$$\begin{array}{l} X_{i}^{q}:=X_{i}^{q}+\left(A_{i}\cdot X^{s}\right)\cdot W_{p}+b_{p} \end{array}$$ (3)

where $X_{i}^{q}\in {\mathbb{R}}^{1\times d}$ is the query feature vector (at query location *i* inside the flattened *HW*×*d* tensor), $A_{i}\in {\left[0,1\right]}^{1\times SHW}$ is the attention map for query location *i*, *X*^*s*^∈ℝ^*SHW*×*d*^ is the support features tensor and $W_{p}\in {\mathbb{R}}^{d\times d}$, $b_{p}\in {\mathbb{R}}^{1\times d}$ are the parameters of a projection layer.

The height *H* and width *W* double from one decoder stage to the next, therefore the coarse attention map must also be upsampled from size (*HW*×*SHW*) to (2*H* 2*W*×*S* 2*H* 2*W*). However, recomputing an upsampled *A* using Equation 2 becomes very expensive for larger *H, W* because the cost scales quadratically with *H*·*W*. The coarse attention map computed by the *Cross Attention Mapper* has an output stride of 32 (i.e., size 8·8 × *S*·8·8), therefore its computation cost is minimal. To maintain computational and memory costs low, we introduce an efficient *Attention Upsampler* block, as follows:

1. The total attention mass is computed for each query position by summing *A* on its last axis, yielding $A_{\text{mass}}\in {\left[0,1\right]}^{HW\times 1}$.
2. *A* is upsampled from (*HW*×*SHW*) to (*HW*×*S* 2*H* 2*W*) using regular interpolation, by first reshaping to (*HW*×*S*×*H*×*W*) and then considering the first and second dimension as the batch and channels axes, respectively. A learnable mix of spatial nearest-neighbor and bilinear interpolation is used: *cI*_nn_+(1−*c*)*I*_lin_, where *c*∈[0, 1] is a learnable parameter and *I*_nn_, *I*_lin_ are the resulting tensors from the two interpolation methods.
3. *A* is upsampled from (*HW*×*S* 2*H* 2*W*) to (2*H* 2*W*×*S* 2*H* 2*W*) using a similar procedure as above, but first transposing *A*. After the interpolations, *A* is transposed back to its initial axes order.
4. The attention mass *A*_mass_ is upsampled using a similar procedure as above to shape (2*H* 2*W*×1). The *A* matrix is normalized so that its sum across all support locations equals *A*_mass_. This step is done to preserve the support set relevance scores w.r.t. each query location, as computed in Section 3.1.1.
5. Based on the upsampled *A*, for each query location *i* the top-*K* most relevant support locations (by sorting decreasingly the attention scores) are extracted from *A*_*i*_ and refined individually using a feed-forward network; its input consists of a concatenation of attention value *A*_*ij*_, the query feature vector of location *i* in the query image and the support feature vector of location *j* in the support set. The query and support feature tensors are selected from the corresponding stages of the pyramid computed by the *Attention Mapper Encoder* and its EMA counterpart, respectively.
6. The feed-forward network's output *y*_FFN_ is used to refine the attention score: *A*_*ij*_ = *A*_*ij*_(1+σ(*y*_FFN_)), where σ is the ELU activation. The factor $\left(1+\sigma \left(y_{\text{FFN}}\right)\right)\in {\mathbb{R}}^{+}$ can either increase or decrease the initial upscaled attention score *A*_*ij*_ based on the learned FFN mapping between query and support local features.
7. Lastly, *A* is re-normalized as in step 4.

The *Attention Upsampler* block refines the attention values for only *K* support set locations that were previously identified as relevant for a query location *i*, thereby optimizing computation. The upscaled attention maps produced by this process remain highly descriptive and do not introduce any unwanted artifacts as long as *K* is large enough. The reasoning is that support set locations with initially low attention values (relative to query location *i*) do not require further refinement after regular interpolation, as low semantic similarity between a query and a support location (indicated by a low attention value) is unlikely to change significantly by doubling the scale of the feature maps. Since transitioning from one decoder stage to the next quadruples the number of locations in the subsequent feature maps (as the *H, W* sizes are doubled), in order to maintain the same ratio of refined attention values the *K* number is also increased by 4 × at each decoder stage that employs *Attention Upsampler* blocks.

**Figure 2:**
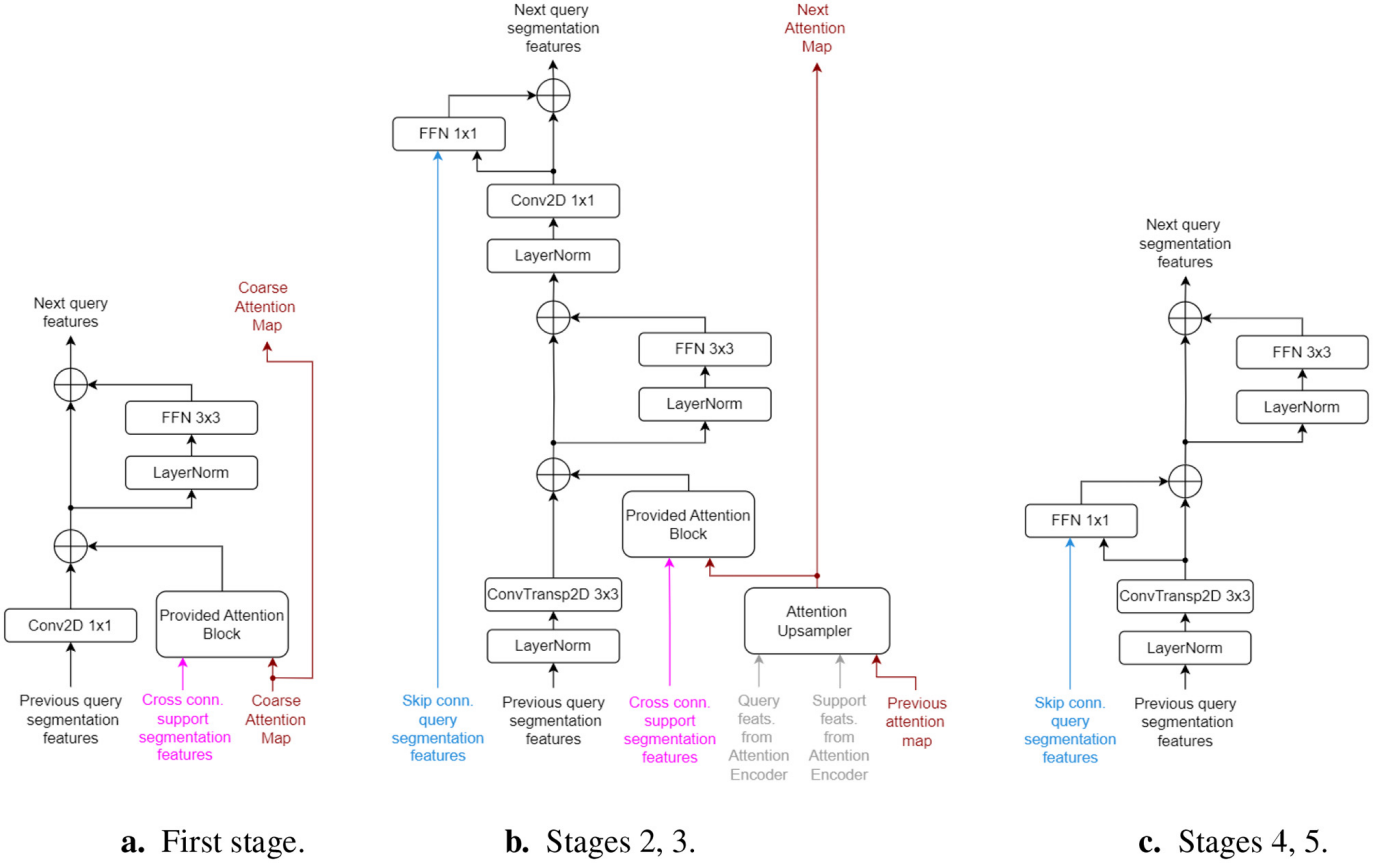
Architecture of *Segmentation Decoder* stages. Inputs have the same colors as in Figure 1. “Cross conn.” signifies cross connections from the support set encoder. **(a)** First stage. **(b)** Stages 2, 3. **(c)** Stages 4, 5.

Figure 2 illustrates the structure of the 5 decoding stages. The first stage directly uses the coarse attention map from the *Cross Attention Mapper* block, as they share the same output stride (i.e., 32 × ). The second and third decoding stages each use an *Attention Upsampler* block, successively upscaling the attention map *A* from one stage to the next. Beginning with the second stage, the query features are upscaled using transposed convolution layers with kernel size of 3 and stride of 2, ensuring compatibility with the regular and cross-support skip connections. All decoder stages follow a residual architecture to maintain adequate gradient flow. The parameters of the last layer in all inner residual blocks are initialized to zero, acting as an identity function at the start of training. All LayerNorm blocks perform z-score normalization across the channel, height, and width axes together, independently for each sample in the batch or support set.

As only four decoder stages perform 2 × upscaling on the query features, an additional final stage (without skip connections) is used to upsample the features to the original input image size. The last layer of this stage is a convolutional projector that outputs a single channel (the predicted mask) with a sigmoid activation. All skip connections, originating from the query, support set, and attention encoders, come from LayerNorm layers, ensuring they are already normalized when entering the decoder stages.

### 3.2 Datasets

To evaluate our model, we used a combination of public and proprietary datasets that span various modalities, avoiding the generation of additional synthetic data as previously done (Butoi et al., 2023). For conciseness, we present a detailed overview of the datasets in Supplementary Table S2. This table includes 9 modalities and totals approximately 438K slices for all tasks (considering that slices with more segmentation masks are used multiple times). The dataset is significantly smaller than those used for tasks such as medical segment anything (Huang Y. et al., 2024; Zhu J. et al., 2024), while it matches in terms of the number of modalities.

To pre-process the datasets and extract labels, we considered for each dataset, an independent organ segmentation as a task. For example, using the term *view ID*, which refers to the label of an organ of interest within the ground-truth volume, to obtain the mask for *view ID 2*, we consider only the annotated pixels/voxels labeled *2*. This is consistent with previous work (Butoi et al., 2023). To tackle the issue of under-represented combinations of datasets and view IDs, we used dataset/task resampling, which ensures a more balanced final training set.

To compile a testing set, we randomly sampled up to 300 independent annotated samples from the splits of the inner datasets, which represent more than 10% from most sets. For the final validation set, we followed a similar approach, sampling either 90 or 180 samples to ensure a balanced representation of imaging modalities. For datasets consisting of 3D volumes, we used transverse (axial) slicing and retained only those slices with at least a 10-pixel area of the target mask, maintaining a resolution of 256 × 256.

Furthermore, we reserved 23 independent tasks exclusively for testing, to ensure that the evaluation accurately reflects the model's capability to follow the support set for segmentation.

### 3.3 Training setup

To train the model, we used the AdamW (Loshchilov and Hutter, 2019) optimizer for 155k steps with a base learning rate of 10^−4^ and an *Attention Upsampler* top-*K* value of 5 for the first 125k steps and a top-*K* value of 24 for the remaining 30k steps. We implemented a step-wise learning rate scheduler that reduces the learning rater by half at 75k and 100k steps, respectively, and a weight decay of 10^−3^.

For baseline comparisons, we retrained the Universeg model (Butoi et al., 2023), which is one of the most robust baselines across modalities and views. To ensure compatibility with our proposed model in the input size and model parameter count, we performed the following changes on the baseline model: we increased the input size from 128 × 128 to 256 × 256 and added a new encoder/decoder stage to retain the size of the inner bottleneck feature map. Additionally, we increased the network width (i.e., number of filters at each layer) to achieve a total number of parameters similar to our proposed model, approximately 25M. This is significantly smaller than methods such as MedSam, where the base models have more than 90M parameters.

Both models were trained using the same train/validation sets and configuration, including a support set size of *S* = 16. The loss function employed was a weighted sum of DICE (using the squared denominator variant proposed in Milletari et al. (2016), with a weight of 1) and binary cross-entropy (with a weight of 0.25). We monitored the validation loss every 5K steps to check if the models were still improving, and used early stopping to select the best models.

Furthermore, we used data augmentation techniques similar to Butoi et al. (2023): vertical and horizontal flipping, rotations, contrast change, intensity flipping, blurring, adding Gaussian noise and using the mask edge as target, with each augmentation *a* being applied with a probability *p*_*a*_ during training. We employed the PyTorch Lightning training framework for easy multi-GPU scaling through DDP. Training of each model took approximately 3 days using 4 NVidia RTX A4500 GPUs.

## 4 Results

### 4.1 Quantitative model analysis

In all testing scenarios, we configured the base top-*K* number to 8 for the most relevant (coarse) locations in the *Attention Upsampler* blocks of the proposed model. This setup resulted in a refinement process for 32 and 128 attention map locations at the decoder stages with output strides of 16 and 8, respectively.

We define a *task* as a combination of a dataset and a *view ID*, as detailed in Supplementary Table S2. A dataset with multiple *view IDs* indicates that several organs were annotated, each with a label corresponding to its *view ID*. To assess a model's overall segmentation performance, we first averaged the DICE scores across the query test samples for each individual task. We then calculated a global DICE score at the task level by averaging across all tasks listed in Supplementary Table S2, and at the dataset level by averaging across all tasks within each dataset and subsequently across all datasets. This latter global DICE score is particularly useful in addressing the variability in the number of tasks across different datasets. We employed the simple DICE score as our metric:


$$\begin{array}{l} \text{DICE}\left(p,t\right)=\frac{2\sum_{i=1}^{HW}p_{i}\cdot t_{i}}{\sum_{i=1}^{HW}p_{i}+\sum_{i=1}^{HW}t_{i}} \end{array}$$ (4)

where *HW* is the total number of pixels in the query image, *p*_*i*_ and *t*_*i*_ are the predicted and target mask value at pixel location *i*, respectively. For each testing query image, we randomly sampled other pairs of (image, mask) to form a fresh support set; this repeated sampling procedure for the support set avoided any possible biases induced by a static choice of the support samples.

**Figure 3:**
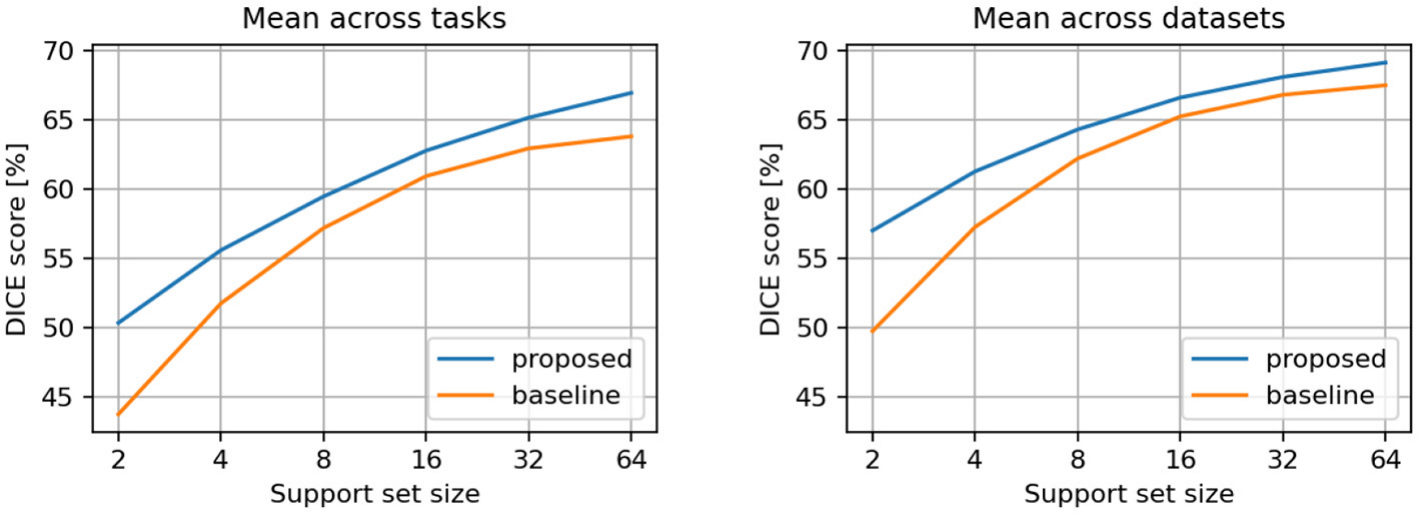
Comparison between proposed and baseline models. Results are averaged across all individual tasks **(left)** and across all individual datasets **(right)**.

The averaged results per tasks and datasets are presented in Table 1 and illustrated in Figure 3. We observed that the proposed model demonstrates superior DICE scores across all support sizes in both aggregation scenarios. Notably, the performance gap widens with smaller support sets, as the baseline model's performance declines faster when smaller support set sizes are used.

**Table 1:** DICE scores [%] for conditional semantic segmentation, across all tasks and datasets.

**Support set size**	**Mean across tasks**		**Mean across datasets**	
	**Proposed**	**Baseline**	**Proposed**	**Baseline**
2	50.37	43.77	57.04	49.78
4	55.62	51.78	61.31	57.30
8	59.50	57.22	64.34	62.24
16	62.81	60.97	66.64	65.28
32	65.19	62.97	68.13	66.84
64	66.98	63.84	69.17	67.53

**Figure 4:**
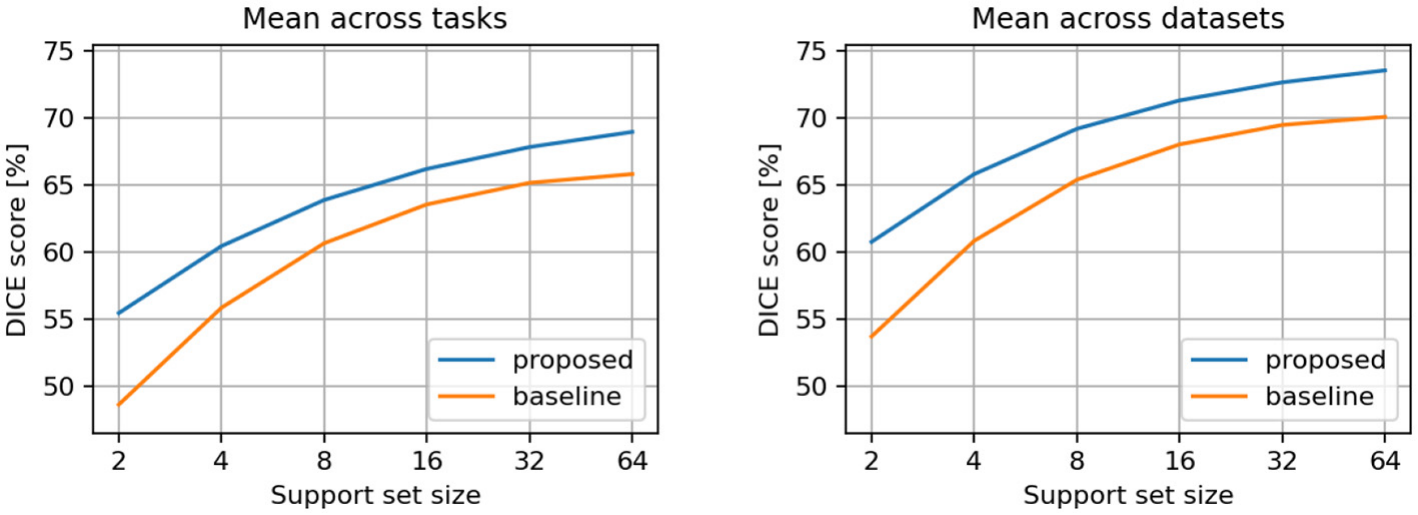
Comparison between proposed and baseline models. Results are averaged across test-only tasks **(left)** and datasets **(right)**.

Additionally, we computed the metrics using only the tasks that were not involved in the training process (i.e., the tasks reserved exclusively for testing, as indicated in Supplementary Table S2). We applied the same aggregation methods as above. The metrics for these test-only tasks are presented in Table 2 and illustrated in Figure 4. The previously observed improvements remain consistent, but the differences between the proposed and baseline models are more pronounced, indicating that the proposed model generalizes better to new tasks.

**Table 2:** DICE scores [%] for conditional semantic segmentation, considering only the testing tasks and datasets.

**Support set size**	**Mean across tasks**		**Mean across datasets**	
	**Proposed**	**Baseline**	**Proposed**	**Baseline**
2	55.45	48.62	60.76	53.70
4	60.45	55.85	65.82	60.84
8	63.89	60.66	69.20	65.40
16	66.21	63.56	71.32	68.04
32	67.84	65.17	72.67	69.49
64	68.97	65.82	73.56	70.09

**Figure 5:**
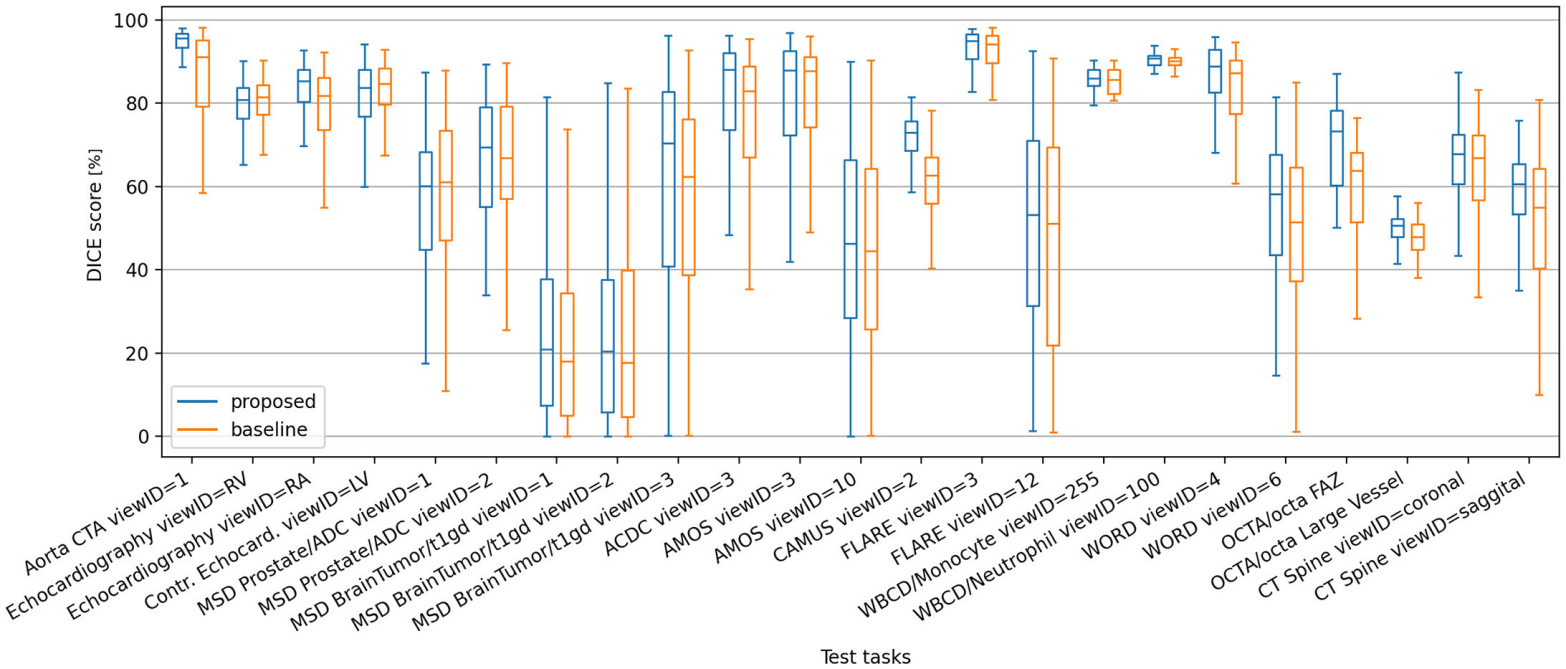
Box plot of DICE scores of the proposed and baseline models on the test-only segmentation tasks, using support set size *S* = 16.

Figure 5 shows a box plot describing the distribution of DICE values on all test-only tasks, using a support set size *S* of 16 (i.e., the same *S* size used during training). Some difficult tasks yield a large spread of DICE scores for both models, however the proposed model exhibits higher metric means and quartile limits in the majority of such tasks. In tasks where the baseline model has consistent performance with narrower spreads of DICE scores, the proposed model behaves at least as good. The same trend of higher DICE means yielded by the proposed model holds also at dataset level, as depicted in Supplementary Figure S1 and Supplementary Table S1.

**Figure 6:**
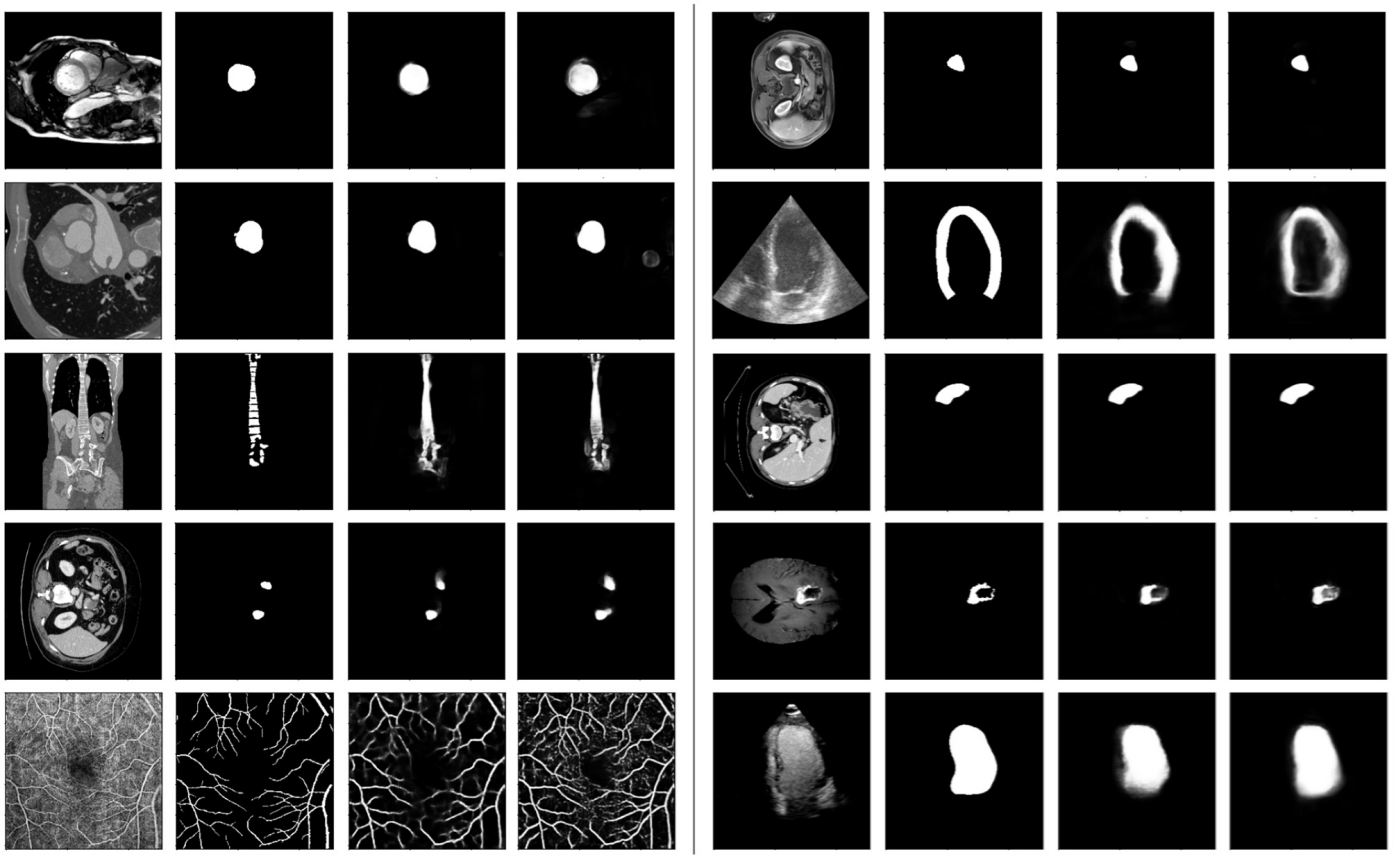
Some samples from the test-only tasks. Each group of 4 sub-plots show the query image, the ground-truth mask, the proposed and baseline model predictions, respectively. For each example, both models had the same support set (not shown).

Figure 6 illustrates a selection of randomly sampled test images, along with their corresponding target masks and predictions from both models. We observe that the proposed model demonstrates superior granularity in capturing smaller structures and excels in segmenting fine details. For example, in the last row, the vessel structures are more accurately delineated by the proposed model.

Overall, we observe a consistent improvement in performance over the baseline method across the evaluated tasks and datasets. Notably, the performance gap between our approach and the baseline becomes more pronounced as the support set size decreases (Figures 3, 4), revealing a robustness advantage of the proposed attention-based in-context segmentation method. We attribute this superior performance at small support set sizes to several complementary mechanisms inherent to the attention-based architecture. First, the attention mechanism enables dynamic, query-dependent weighting of support features, allowing the model to identify and prioritize the most discriminative characteristics even when only 2 examples are available. Second, at very small support sizes, individual examples may contain idiosyncratic features or annotation artifacts that disproportionately influence the baseline's predictions. The proposed approach mitigates this risk through its attention-based weighting mechanism, which distributes influence across multiple spatial locations. This implicit regularization prevents the model from over-committing to potentially misleading cues present in any single support image, thereby maintaining robustness at small support set sizes.

Comprehensive ablation studies regarding the choice of architectural decisions and hyperparameters are further provided in Section 4 of the Supplementary material.

### 4.2 Qualitative model analysis

To further analyze the model, we examined its attention maps for random test queries using a support set size of 8. Supplementary Figures S2, S3, S4 illustrate the attention maps of a query location, marked by a red indicator, relative to all locations within the support set at the first three stages of the decoder. These attention maps are up-sampled to match the dimensions of the query image. The first stage's attention map is the least detailed (output stride 32), focusing on larger areas across multiple support samples. This stage does a general region-to-region mapping, while the subsequent two attention stages become more specialized, concentrating on smaller regions that exhibit visual and semantic similarity to the marked query location. These stages enable the model to segment finer details and better adhere to the contours of the target organ.

We conjecture that the attention maps produced by the model, given the architectural decisions detailed earlier, enable model explainability by design. To further explore this property, we compare the maps with post-hoc explainability methods. For models trained on medical imaging data, CAM (Class Activation Map) techniques have been widely adopted (van der Velden et al., 2022). Notably, Score-CAM (Wang et al., 2020) and LayerCAM (Jiang et al., 2021) are highly regarded methods. Although they were not originally designed for conditional segmentation models, they can be adapted for this purpose.

We focus on explaining the predicted mask logit for a single query image pixel, using the support set images to construct visual explanations. CAM-based methods require selecting specific inner model layers to generate feature maps. For the baseline model, we use support set features from the last two encoder stages and all decoder stages, as they should hold sufficient information given their architecture. For the proposed model, we use the first three levels of the feature pyramid computed by the *Support Set Encoder*, as these levels are influenced by the attention maps and represent the only information pathways from the support set target masks.

The Score-CAM method constructs masks using all channels from selected feature maps to modulate the input image. For each modified input, a forward pass is necessary to assess its impact on the model output. A score is calculated for each channel from the chosen feature maps, which is then used to weight and combine all activation maps. However, this approach can become computationally intensive, especially with larger feature maps, multiple layers, and larger support sets, as the number of required forward passes increases significantly. This makes Score-CAM impractical for real-time explainability. Therefore, we use the LayerCAM method in this study, which requires only one additional backward pass. LayerCAM employs thresholded gradients ($g_{ij}^{\text{th}}:=0$ if *g*_*ij*_ < 0) to modulate activation maps, summing them along the channel dimension and retaining only positive contributions from each layer. The resulting maps from multiple layers are up-sampled to the input image size and merged using a pixel-wise maximum operation.

**Figure 7:**
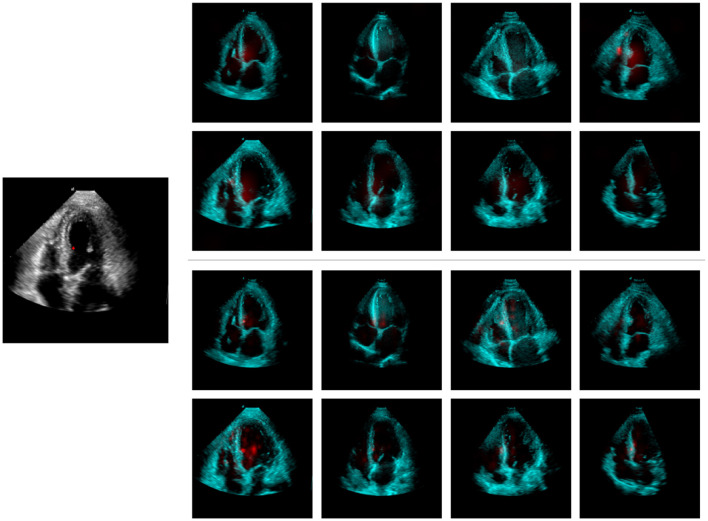
LayerCAM explainability maps for the proposed **(top-right)** and the baseline **(bottom-right)** models; the maps have been computed w.r.t. the query image **(left)** marked location. Support set target segmentation masks are not shown.

Figure 7 illustrates the LayerCAM visual explanation maps for both the proposed and baseline models, using the same query and support set as in Supplementary Figure S2. When comparing the LayerCAM explanations with the attention maps generated by our model (Supplementary Figure S2), some similarities can be observed. However, the CAM approach introduces artifacts by assigning significant weight to irrelevant areas, such as the right ventricle free-wall of the fourth support image, which were not highlighted by our model's attention maps. Given the highly nonlinear nature of neural networks, gradient-based explanation methods may be susceptible to noise. Once the model has sufficient evidence from the support set to account for most of the predicted logit value, further variations in this value may be minor and influenced by factors unrelated to the predicted outcome. This phenomenon has been observed and studied in previous research (Danu et al., 2020).

Given this comparison, the proposed model can be considered inherently explainable, eliminating the need for additional post-hoc methods and avoiding further approximations and artifacts in the visual explanation maps. Additionally, our three-level attention maps highlight the model's focus at each feature scale, providing deeper insight into its reasoning for constructing the output.

### 4.3 Using image retrieval to improve performance

The proposed model employs an internally computed attention map to weigh the embeddings from all support set locations (i.e., *S* support examples with *H*×*W* locations each, where *H, W* depend on the decoder stage). This mechanism can act as a retrieval system, where the most relevant support embeddings are pooled for each query location. However, this retrieval process can be expanded beyond the current support set to include many more relevant samples. For example, to improve the segmentation of a region of interest in the query image, its local embedding (generated by the *Attention Mapper Encoder*) can be used to search within a larger collection of annotated samples. This search can be used to identify images with regions highly similar to the query. The closest matches can then form a new support set, which should yield improved segmentation for the query region of interest.

To evaluate the potential impact of this dynamic support set selection mechanism, we designed the following experiment: For a new task *T* consider an array of labeled samples ${\text{D}}_{s}^{T}=\left\{\left(i_{1},m_{1}\right),...,\left(i_{N},m_{N}\right)\right\}$ and an array of unlabeled samples ${\text{D}}_{q}^{T}=\left\{i_{1},...,i_{M}\right\}$, where (*i, m*) represents an image and its corresponding ground-truth mask, *N* and *M* are the number of labeled and unlabeled images, respectively. For each unlabeled sample *i*_*q*_, a mask *m*_*q*_ must be generated using the annotated samples: $m_{q}|i_{q},{\text{D}}_{s}^{T}$. Training a standalone segmentation algorithm on ${\text{D}}_{s}^{T}$ and predicting on samples from ${\text{D}}_{q}^{T}$ is likely to suffer from overfitting when *N* is very small (e.g., a few hundred samples or even fewer). In such cases, a conditional segmentation model may be more effective, with ${\text{D}}_{s}^{T}$ serving as source of support samples. However, building the required support set raises a challenge: not all *N* samples from ${\text{D}}_{s}^{T}$ can be simultaneously used due to computational constraints. If a fixed support set size *S* is to be used (similar to the experiments above), then *S* samples must be chosen from ${\text{D}}_{s}^{T}$. A static choice (use the same *S* support samples to predict the entire ${\text{D}}_{q}^{T}$) may be sub-optimal. We investigate if extending the functionality of the *Cross Attention Mapper* across the entire ${\text{D}}_{s}^{T}$ leads to a boost in segmentation DICE scores for the ${\text{D}}_{q}^{T}$ set. For this task, we used the test-only tasks of Tab. S2 within the defined scenario. We set *N* = 150 and *M* = 100,^1^ following the criteria that these counts should be: (i) realistic under a low data regime, (ii) small enough (relative to the amount of available data) to allow sampling of multiple instances of ${\text{D}}_{s}^{T}$ and ${\text{D}}_{q}^{T}$ with enough variability between sampled instances, and (iii) large enough to limit the confidence interval when computing the average DICE increment. We applied the EMA version of the *Attention Mapper Encoder* on all samples from ${\text{D}}_{s}^{T}$ and saved for each one the coarsest feature map, on which we applied a projection layer equivalent to the projections done inside the attention layer of the *Cross Attention Mapper*. The resulting vectors were stored in a structure $D_{S}$.

Due to the repeated sampling required in our analysis, we used a Monte Carlo approach to estimate the impact of using a custom support-set w.r.t. each query image vs. using a static generic one. We followed these steps in our analysis:

1. randomly choose *S* samples from ${\text{D}}_{s}^{T}$ and create a static and generic support set.
2. for all query images from ${\text{D}}_{q}^{T}$:

(a) predict a mask using the generic support set, compute its DICE score and sample some locations around its contour.
(b) For the sampled contour locations extract their *Attention Encoder* embeddings and search $D_{S}$ for their nearest neighbor vectors (using the embeddings cross-correlation value as similarity metric, as done inside the softmax of Equation 2); use the matched images from $D_{S}$ to form a custom support set.
(c) Predict a new mask for the current query using the custom support set; compute the DICE score.
(d) compute the increment Δ in DICE score when switching from the generic to the custom support set.

3. average Δ across all tasks and datasets.

We executed the above procedure 10 times and averaged the results. For each iteration, we randomly resampled the ${\text{D}}_{s}^{T}$ and ${\text{D}}_{q}^{T}$ arrays. Table 3 displays the improvements in DICE scores when querying ${\text{D}}_{s}^{T}$ for the best support images for each query image, compared to using a fixed and generic support set. The improvements are more significant with smaller support set sizes, where limited sample variability and uninformative support samples can negatively impact the final prediction.

**Table 3:** DICE score increments Δ (considering only the testing tasks and datasets) when using retrieval-based custom support set.

**Support set size**	**Mean** Δ **across tasks**		**Mean** Δ **across datasets**	
	**Proposed**	**Baseline**	**Proposed**	**Baseline**
4	4.70	6.11	4.16	6.17
8	3.41	4.34	3.07	4.42
16	2.67	3.71	2.38	3.84

We also evaluated this approach on the baseline model to determine if the DICE score improvements apply to unrelated models. Since the baseline model experienced even larger DICE score increases, this further supports the effectiveness of the proposed attention mapping mechanism for retrieval.

## 5 Conclusions

We introduce a universal few-shot conditional semantic segmentation framework for medical imaging based on the principle that selective attention to relevant support set regions enhances segmentation accuracy. Our approach implements cross-attention mechanisms between query and support images, integrated with an efficient attention upsampling strategy that computes correspondences at reduced feature scales before hierarchical refinement to higher resolution. This design inherently supports explainability through direct visualization of support-query spatial relationships.

Comprehensive evaluation across 29 medical datasets spanning 9 imaging modalities and 135 segmentation tasks demonstrates superior performance and improved robustness compared to baseline methods (Butoi et al., 2023). Critically, our approach exhibits greater robustness at minimal support set sizes (S = 2–4), where baseline performance degrades substantially. These advantages are achieved while preserving interpretability and enabling query-adaptive support set customization for prediction refinement. Specifically, the method enables content-based image retrieval to identify the most semantically similar annotated samples from an existing database, thereby constructing a query-personalized support set with improved representativeness for the target segmentation. The similarity metrics underlying this retrieval process are computed directly from our architecture via a *Cross Attention Mapper* module, which establishes dense spatial correspondences by mapping each query location to all contextually relevant positions across the support set samples. This retrieval-based selection mechanism yields further performance improvements compared to random support set sampling.

We believe the proposed architecture advances the development of robust foundational models for medical image segmentation with flexible conditioning mechanisms that support diverse input modalities. Promising avenues for future work include extending the framework to 3D volumetric inputs, leveraging the computational efficiency of the attention upsampling module to maintain tractable memory and runtime requirements despite the increased dimensionality. Specifically, for 3D volumetric inputs the primary bottleneck is the quadratic memory scaling inherent to the attention mechanisms. Nevertheless, our Attention Upsampling architecture is inherently well-suited to address this scalability challenge. The key insight is the decoupling of the attention computation from spatial resolution: cross-attention is performed exclusively on low-resolution encoder features, where the spatial dimensions are reduced through downsampling. The resulting attention maps are then upsampled to provide guidance for higher-resolution decoder layers. This strategy significantly reduces memory requirements while enabling the model to leverage support set information throughout the decoding process. This computational efficiency makes 3D extension practical while preserving the model's few-shot learning capabilities.

## Data Availability

The original contributions presented in the study are included in the article/Supplementary material, further inquiries can be directed to the corresponding author.

## Footnotes

1 For this use case, we extended the amount of data for some of our test-only tasks to more than what is shown in Supplementary Table S2.
