# Supplementary material for: Universal medical image segmentation via in-context cross-attention
Source: Front Artif Intell. 2025 Nov 25;8:1698324. doi: 10.3389/frai.2025.1698324 (PMC12685931; doi:10.3389/frai.2025.1698324)
Supplement: Supplementary file 1 [file Data_Sheet_1.pdf]

Supplementary Material

1 EXTENDED RESULTS

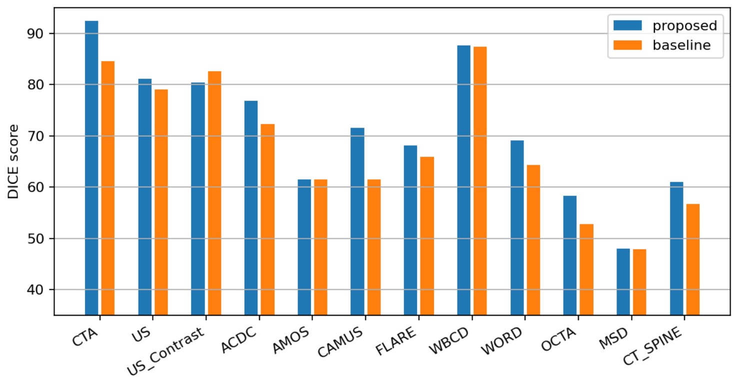

Figure S1: Bar plot of mean DICE scores of the proposed and baseline models on the test-only segmentation tasks aggregated by datasets, using support set size $S = 16$. "CTA" and "US" are abbreviations for "Aorta_CTA" and "Echocardiography", respectively.

Table S1. Average DICE scores for the test-only tasks, aggregated at dataset-level, for all considered support set sizes. "BL" means baseline model. "US" is abbreviation for "Echocardiography".

Dataset	S=2		S=4		S=8		S=16		S=32		S=64	
	ours	BL	ours	BL	ours	BL	ours	BL	ours	BL	ours	BL
Aorta_CTA	75.88	61.43	84.85	74.70	90.30	80.86	92.40	84.62	92.95	86.43	93.10	87.16
US	74.56	69.46	78.77	74.97	80.40	77.80	81.12	79.05	81.03	79.80	81.02	80.10
US_Contrast	74.30	76.07	77.43	79.89	79.38	81.71	80.35	82.59	80.74	83.16	80.88	83.36
ACDC	67.52	56.11	73.18	65.65	75.07	70.09	76.83	72.26	78.01	73.56	79.28	74.49
AMOS	46.65	42.50	52.37	50.62	57.48	57.13	61.45	61.48	64.34	63.90	67.46	65.31
CAMUS	63.54	56.56	67.82	58.89	70.62	61.03	71.57	61.54	71.74	61.66	71.33	61.46
FLARE	50.10	41.88	56.36	52.25	61.40	58.81	68.15	65.88	73.91	70.20	77.01	71.65
WBCD	86.88	80.26	87.37	84.98	87.67	86.75	87.63	87.38	87.48	87.66	87.48	87.66
WORD	47.61	38.69	57.10	49.06	65.18	60.08	69.07	64.26	71.72	66.87	74.08	68.20
OCTA	53.84	44.90	56.23	49.14	57.82	51.20	58.33	52.76	58.65	52.87	58.79	52.80
MSD	34.65	31.52	40.90	39.26	44.72	44.10	47.94	47.90	50.68	50.02	52.76	50.58
CT_SPINE	53.55	45.00	57.42	50.71	60.32	55.26	61.03	56.71	60.83	57.71	59.48	58.36

2 DATASETS

Table S2: Breakdown of datasets used in this study. The colored entries were used solely for testing. Datasets that contained multiple organ-centric subsets or modalities are represented in the second column as ”MainName / SubSetName / Modality”.

Dataset type	Dataset name	view ID	train set size	vld set size	test set size
Private	Coronary Angiography	-	2351	180	300
Private	Aorta_MRI	-	12099	90	300
Private	Aorta_CTA	1	0	0	300
		2	2687	90	300
		3	2687	90	300
Public Graham et al. (2019)	CONSEP	-	24	0	17
Private	Echocardiography	LV	36573	180	300
		LA	16083	180	300
		RV	0	0	164
		RA	0	0	182
Private	Contrast Echocardiography	LV	0	0	300
Public Simpson et al. (2019)	MSD/Liver	1	9000	90	299
		2	5220	90	272
Public Simpson et al. (2019)	MSD/Heart	-	1025	90	198
Public Simpson et al. (2019)	MSD/ Prostate/T2	1	265	32	36
		2	347	44	53
Public Simpson et al. (2019)	MSD/ Prostate/ADC	1	0	0	36
		2	0	0	53
Public Simpson et al. (2019)	MSD/Lung	-	1347	90	123
Public Simpson et al. (2019)	MSD/Pancreas	1	6991	90	293
		2	1942	90	298
Public Simpson et al. (2019)	MSD/Hepatic Vsl.	1	9227	90	233
		2	3930	90	288
Public Simpson et al. (2019)	MSD/Spleen	-	846	90	90

Public Simpson et al. (2019)	MSD/Colon	-	1034	90	121
Public Simpson et al. (2019)	MSD/Hippocampus	1	3137	90	270
		2	4387	90	300
Public Simpson et al. (2019)	MSD/BrainTumor/ FLAIR	1	9000	90	289
		2	9000	90	262
		3	8209	90	285
Public Simpson et al. (2019)	MSD/BrainTumor/ T1w	1	9000	90	289
		2	9000	90	262
		3	8209	90	285
Public Simpson et al. (2019)	MSD/BrainTumor/t1gd	1	0	0	289
		2	0	0	262
		3	0	0	285
Public Simpson et al. (2019)	MSD/BrainTumor/ T2w	1	9000	90	289
		2	9000	90	262
		3	8209	90	285
Public Bernard et al. (2018)	ACDC	1	1390	90	300
		2	1655	90	300
		3	0	0	299
Public Ji et al. (2022)	AMOS	1	4953	90	300
		2	5498	90	299
		3	0	0	298
		4	2198	90	299
		5	5773	90	267
		6	8488	90	299
		7	5771	90	297
		8	8000	90	298
		9	8000	90	297
		10	0	0	298
		11	1802	90	142
		12	2086	90	211
		13	5136	90	299
		14	2523	34	21
		15	2302	23	38
Public Zhang et al. (2022)	BUS	malign	168	21	21
		benign	349	44	44
		normal	103	17	0

Public Leclerc et al. (2019)	CAMUS	1	1680	160	160
		2	0	0	160
		3	1680	160	160
Public Kavur et al. (2021)	CHAOS/CT	-	2300	90	216
Public Kavur et al. (2021)	CHAOS/MR	-	517	65	45
Public Staal et al. (2004)	DRIVE/Disk	-	20	0	20
Public Staal et al. (2004)	DRIVE/Vessel	-	20	0	0
Public Bano et al. (2020)	FETOPLAC	-	387	48	48
Public Ma et al. (2024)	FLARE	1	2272	90	300
		2	1458	90	226
		3	0	0	180
		4	1158	90	172
		5	3134	90	300
		6	2880	90	300
		7	529	63	64
		8	596	69	73
		9	642	68	103
		10	980	90	148
		11	1402	90	193
		12	0	0	189
		13	1487	90	229
Public Hernandez Petzsche et al. (2022)	ISLES	-	3790	90	281
Public Porwal et al. (2018)	IDRID/Haemorr.	-	47	26	0
Public Porwal et al. (2018)	IDRID/H. Exud.	-	43	17	10
Public Porwal et al. (2018)	IDRID/ MicroAneur.	-	32	0	30
Public Porwal et al. (2018)	IDRID/Opt. Disk	-	46	17	18
Public Porwal et al. (2018)	IDRID/S. Exud.	-	21	17	0

Public Litjens et al. (2014)	PROMISE12	-	695	83	300
Public Mazurowski et al. (2017)	LGG	-	1063	90	146
Public Bilic et al. (2023)	LITS	1	5441	90	278
		2	1837	90	173
Public Heller et al. (2021)	KITS	1	9000	90	300
		2	9000	90	298
		3	3954	90	193
Public Setio et al. (2017)	LUNA16	3	10000	90	296
		4	10000	90	297
		5	10000	90	296
Public Wasserthal (2024)	CT Spine	coronal	0	0	299
		saggital	0	0	299
Public Karim et al. (2013)	CDEMRIS	-	1509	90	185
Public Zheng et al. (2018)	WBCD/Basophil	255	158	30	30
Public Zheng et al. (2018)	WBCD/Eosinophil	100	141	30	30
		255	141	30	30
Public Zheng et al. (2018)	WBCD/Lymphocyte	100	182	30	30
		255	182	30	30
Public Zheng et al. (2018)	WBCD/Monocyte	100	182	30	30
		255	0	0	30
Public Zheng et al. (2018)	WBCD/Neutrophil	100	0	0	30
		255	182	30	30
Public Luo et al. (2022)	WORD	1	4180	90	300
		2	2559	90	249
		3	2931	90	300
		4	0	0	300
		5	3248	90	279
		6	0	0	61
		7	2071	90	255
		8	2214	90	205
		9	2537	90	299
		10	7458	90	300
		11	6875	90	300
		12	1228	90	116

		13	2529	90	268
		14	1757	90	206
		15	2316	90	260
		16	2314	90	260
Public Li et al. (2024)	OCTA/FAZ/oct	-	400	50	50
Public Li et al. (2024)	OCTA/Large Vsl./oct	-	400	50	50
Public Li et al. (2024)	OCTA/FAZ/octa	-	0	0	50
Public Li et al. (2024)	OCTA/Large Vsl./octa	-	0	0	50
Public Kuijf et al. (2019)	WMH	1	2363	90	247
Public Abdi et al. (2015)	PANDENTAL	Jaw	92	0	24

3 EXAMPLE ATTENTION MAPS

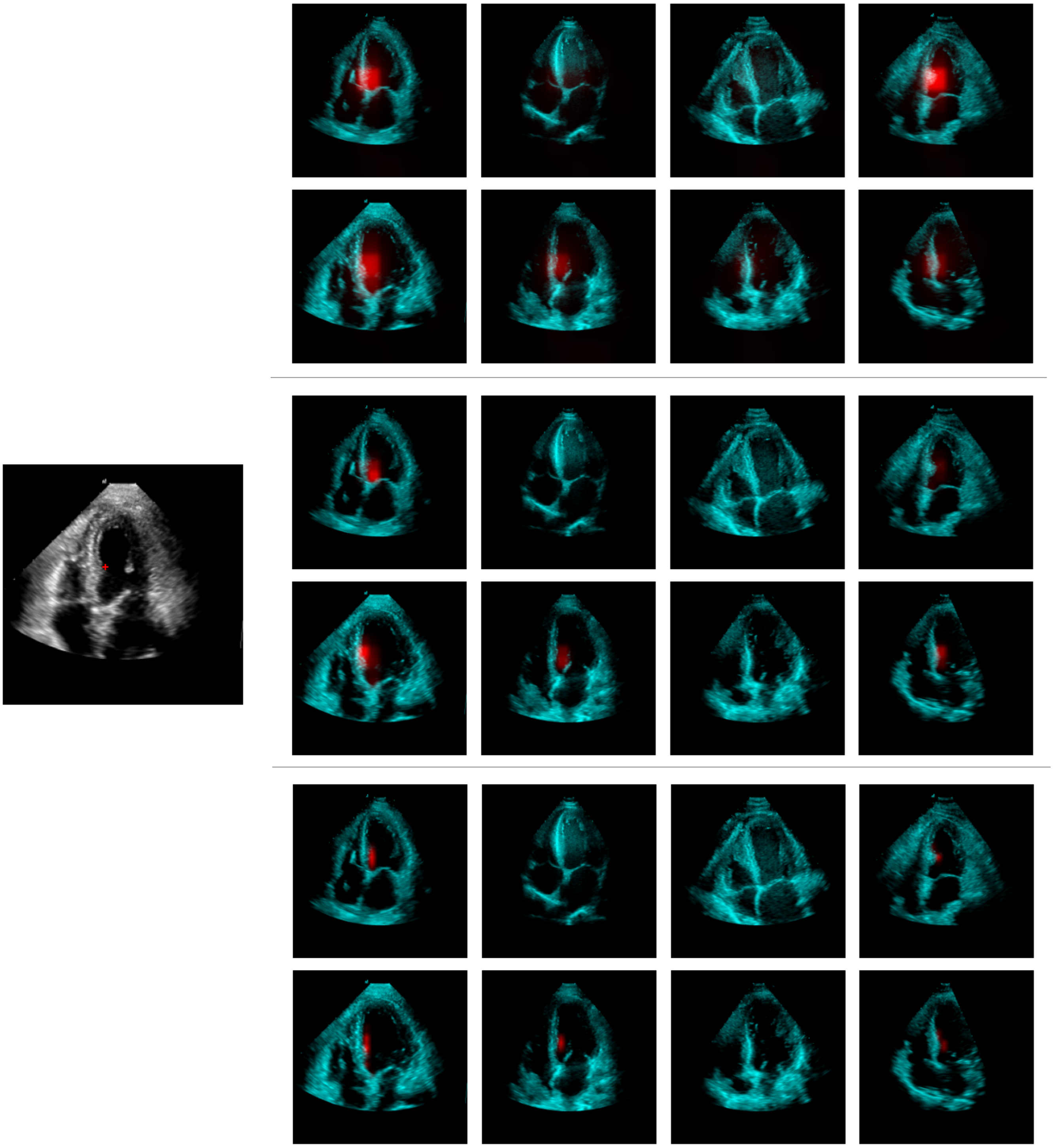

Figure S2: Example attention maps for an echocardiography. The attention maps over the support set from the first 3 decoder stages (right column, from coarse maps at the top grid to fine maps at the bottom grid) are depicted w.r.t. the red query location (left column).

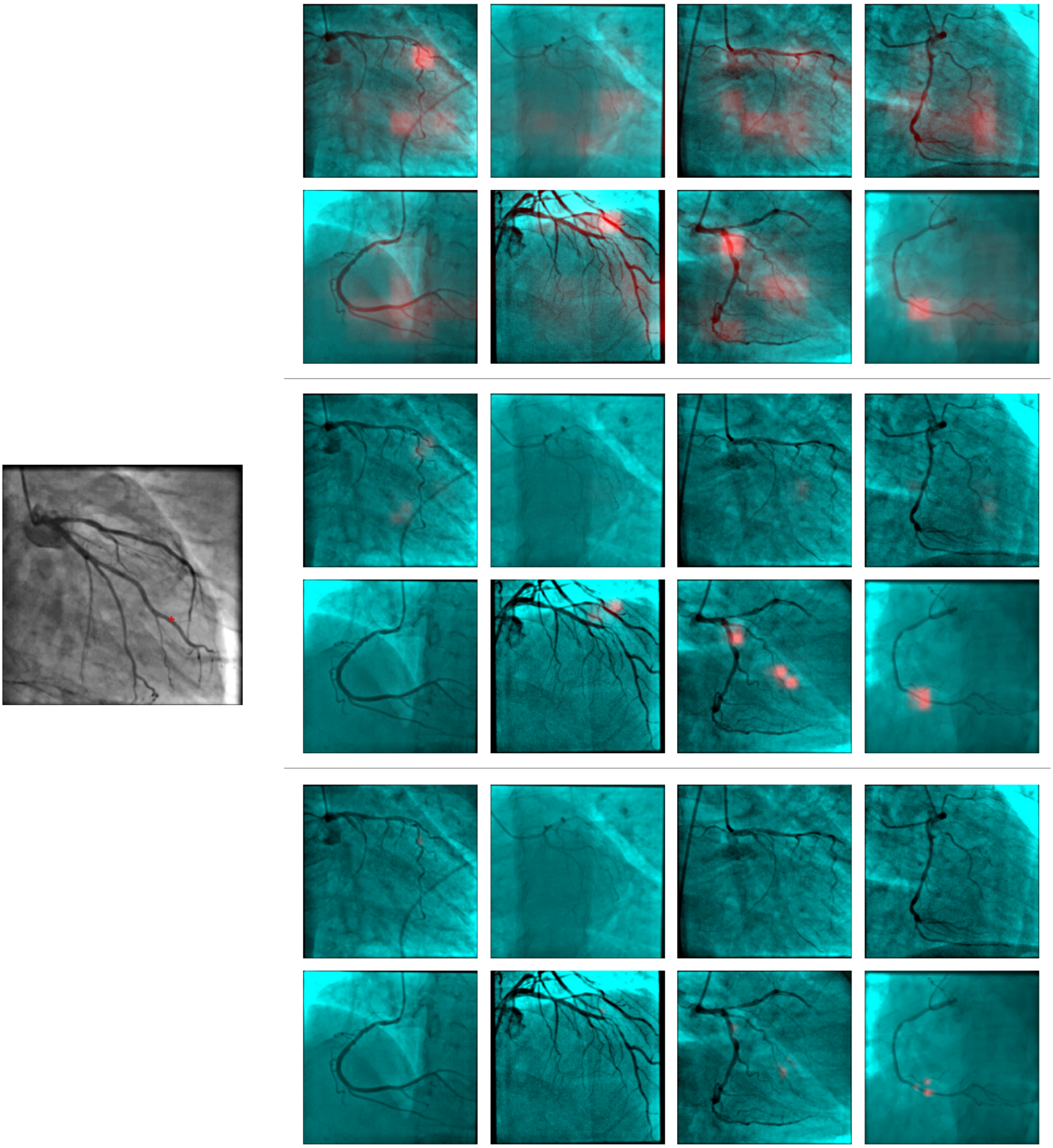

Figure S3: Example attention maps for a coronary angiography. The attention maps over the support set from the first 3 decoder levels (right column, from coarse maps at the top grid to fine maps at the bottom grid) are depicted w.r.t. the red query location (left column).

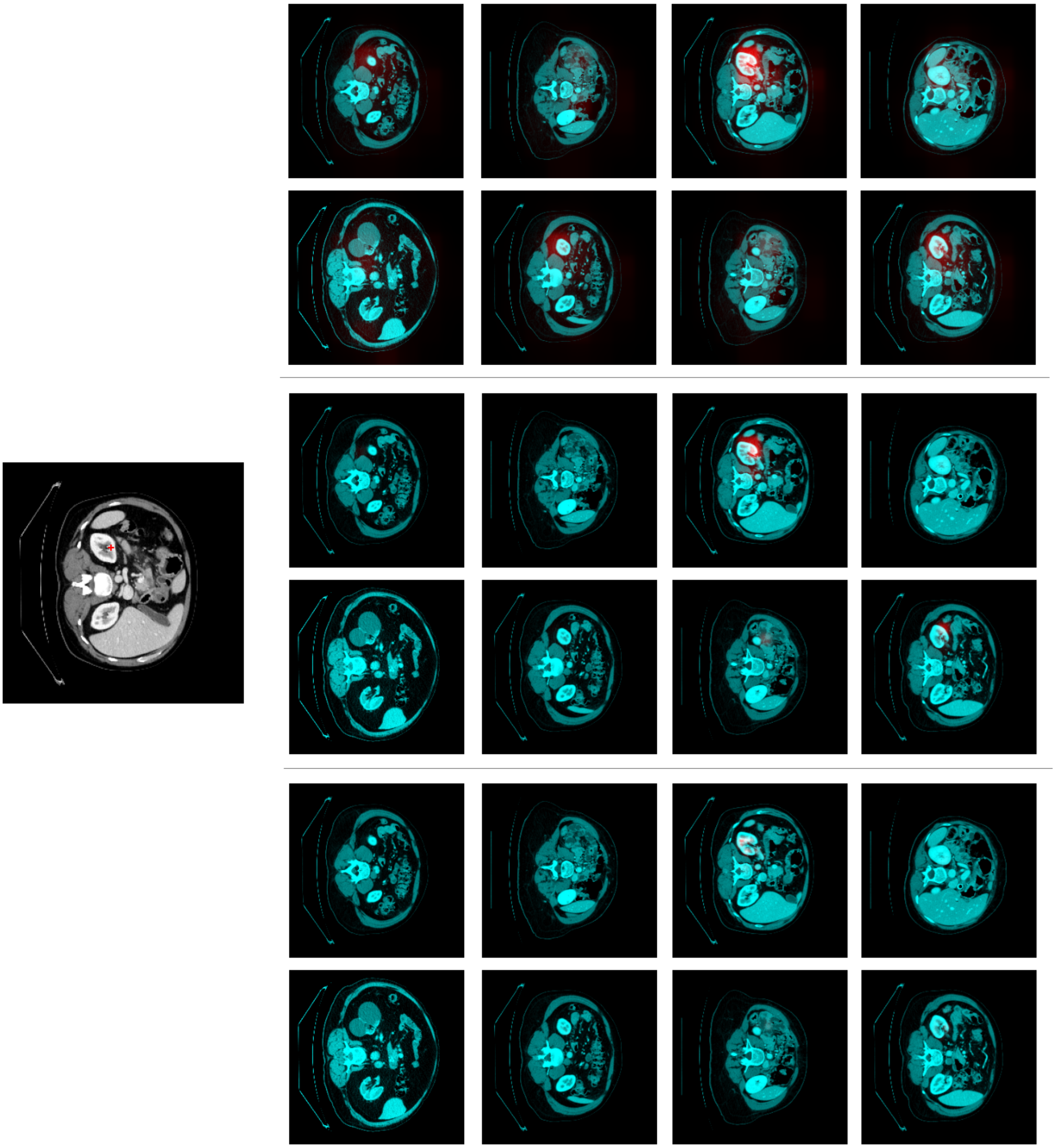

Figure S4: Example attention maps for an abdominal CT. The attention maps over the support set from the first 3 decoder levels (right column, from coarse maps at the top grid to fine maps at the bottom grid) are depicted w.r.t. the red query location (left column).

4 ABLATION STUDIES

This section provides ablation studies and analyses to validate the design choices. We first investigate the $topK$ hyperparameter to identify the optimal balance between segmentation accuracy and inference efficiency. We then evaluate the contribution of our encoder architectural components through controlled ablations. Finally, we quantify computational costs across different support set sizes, comparing our method against both the baseline and non-conditional segmentation models.

4.1 Attention Upsampler

We investigate the influence of the *topK* parameter in the *Attention Upsampler* layers within the *Segmentation Decoder*. Using the trained model described in Sec. 3.3 of the main text, we recomputed test Dice scores across various *topK* values. Since this hyperparameter can be adjusted at inference time without retraining, identifying the optimal trade-off between predictive performance and computational cost is of practical importance.

Tab. S3 presents the effect of varying *topK* with support set size S=16. Computational cost per inference – measured in floating-point operations (GFLOP) – scales linearly with *topK*. Peak Dice scores are achieved at *topK*=16; however, the improvement over *topK*=8 is marginal. Consequently, we adopt *topK*=8 for all experiments reported in the main text, as it provides substantially faster inference with negligible performance degradation.

Table S3. Proposed model's test DICE scores [%] versus the *topK* parameter of the *Attention Upsampler* layers inside the *Segmentation Decoder*. Support set size was fixed at 16 example pairs.

Mean across	*topK* 4	8	16	32
tasks	65.46	66.21	66.31	66.01
datasets	70.60	71.32	71.41	71.11
GFLOP	174.62	177.99	184.74	198.24

4.2 Architectural blocks

We further conducted an ablation study to evaluate the impact of the encoder design choices on test Dice scores. Specifically, we compared the proposed architecture against two alternative configurations, as follows:

1. **Using EMA vs. not using EMA**: This means training without Exponential Moving Average updates. Instead, the same *Attention Mapper Encoder* instance processes both query images and support set images directly;
2. **Increasing the FoV**: Employing standard MSCAN encoders for both the *Segmentation Encoder* and *Support Set Encoder*, rather than the small field-of-view variants described in Sec. 3.1.2 of the main text.

Both alternative configurations were trained and evaluated using the identical protocol described in Sec. 3.3 of the main text. Tab. S4 presents the results, and showcases that the proposed architecture achieves superior test performance. More precisely, the EMA encoder provides stability and improved performances at different support set sizes, while increasing the FoV leads to a worsening in performance.

Table S4. Test DICE scores [$\%$]) of ablation scenarios.

Support set size	Mean across tasks			Mean across datasets		
	proposed	w/o EMA	big FoV	proposed	w/o EMA	big FoV
4	**60.45**	58.87	53.96	**65.82**	64.26	57.51
8	**63.89**	62.50	56.14	**69.20**	67.74	59.71
16	**66.21**	65.00	57.51	**71.32**	70.07	61.05

4.3 Computational Cost Comparison

We further quantified the computational cost (GFLOP) required to generate a prediction for a single query sample as a function of support set size S. For reference, we benchmark the cost against two non-conditional segmentation models—SwinUNETR Hatamizadeh et al. (2021) and residual UNet Kerfoot et al. (2019) – each configured to have a comparable number of parameters to our proposed and baseline models.

Tab. S5 illustrates the GFLOP counts across all models. Both the proposed and baseline conditional models exhibit linear scaling of computational cost with increasing S. However, the baseline model demonstrates a steeper computational growth rate: the GFLOP overhead relative to our proposed method increases from 5.4× at $S = 2$ to 8× at $S = 8$. Notably, at $S = 1$, our proposed model achieves computational efficiency comparable to non-conditional architectures.

Table S5. GFLOP comparison between models and support set sizes. Unconditional segmentors do not rely on a support set, therefore are marked with *na* (not applicable).

Model	GFLOP per inference					
	S=1/none	S=2	S=4	S=8	S=16	S=32
proposed	30.58	40.41	60.07	99.37	177.99	335.23
baseline	121.27	218.91	414.19	804.73	1585.83	3148.02
SwinUNETR	35.9	*na*	*na*	*na*	*na*	*na*
residual UNet	7.12	*na*	*na*	*na*	*na*	*na*

REFERENCES

Abdi, A. H., Kasaei, S., and Mehdizadeh, M. (2015). Automatic segmentation of mandible in panoramic x-ray. *Journal of Medical Imaging* 2, 044003–044003

Bano, S., Vasconcelos, F., Shepherd, L. M., Vander Poorten, E., Vercauteren, T., Ourselin, S., et al. (2020). Deep placental vessel segmentation for fetoscopic mosaicking. In *Medical Image Computing and Computer Assisted Intervention–MICCAI 2020: 23rd International Conference, Lima, Peru, October 4–8, 2020, Proceedings, Part III 23* (Springer), 763–773

Bernard, O., Lalande, A., Zotti, C., Cervenansky, F., Yang, X., Heng, P.-A., et al. (2018). Deep learning techniques for automatic mri cardiac multi-structures segmentation and diagnosis: is the problem solved? *IEEE transactions on medical imaging* 37, 2514–2525

Bilic, P., Christ, P., Li, H. B., Vorontsov, E., Ben-Cohen, A., Kaissis, G., et al. (2023). The liver tumor segmentation benchmark (lits). *Medical image analysis* 84, 102680

Graham, S., Vu, Q. D., Raza, S. E. A., Azam, A., Tsang, Y. W., Kwak, J. T., et al. (2019). Hover-net: Simultaneous segmentation and classification of nuclei in multi-tissue histology images. *Medical image analysis* 58, 101563

Hatamizadeh, A., Nath, V., Tang, Y., Yang, D., Roth, H. R., and Xu, D. (2021). Swin unetr: Swin transformers for semantic segmentation of brain tumors in mri images. In *International MICCAI brainlesion workshop* (Springer), 272–284

Heller, N., Isensee, F., Maier-Hein, K. H., Hou, X., Xie, C., Li, F., et al. (2021). The state of the art in kidney and kidney tumor segmentation in contrast-enhanced ct imaging: Results of the kits19 challenge. *Medical image analysis* 67, 101821

Hernandez Petzsche, M. R., de la Rosa, E., Hanning, U., Wiest, R., Valenzuela, W., Reyes, M., et al. (2022). Isles 2022: A multi-center magnetic resonance imaging stroke lesion segmentation dataset. *Scientific data* 9, 762

Ji, Y., Bai, H., Ge, C., Yang, J., Zhu, Y., Zhang, R., et al. (2022). Amos: A large-scale abdominal multi-organ benchmark for versatile medical image segmentation. *Advances in neural information processing systems* 35, 36722–36732

Karim, R., Housden, R. J., Balasubramaniam, M., Chen, Z., Perry, D., Uddin, A., et al. (2013). Evaluation of current algorithms for segmentation of scar tissue from late gadolinium enhancement cardiovascular magnetic resonance of the left atrium: an open-access grand challenge. *Journal of Cardiovascular Magnetic Resonance* 15, 105

Kavur, A. E., Gezer, N. S., Barış, M., Aslan, S., Conze, P.-H., Groza, V., et al. (2021). Chaos challenge-combined (ct-mr) healthy abdominal organ segmentation. *Medical image analysis* 69, 101950

Kerfoot, E., Clough, J., Oksuz, I., Lee, J., King, A. P., and Schnabel, J. A. (2019). Left-ventricle quantification using residual u-net. In *Statistical Atlases and Computational Models of the Heart. Atrial Segmentation and LV Quantification Challenges* (Springer International Publishing), 371–380

Kuijf, H. J., Biesbroek, J. M., De Bresser, J., Heinen, R., Andermatt, S., Bento, M., et al. (2019). Standardized assessment of automatic segmentation of white matter hyperintensities and results of the wmh segmentation challenge. *IEEE transactions on medical imaging* 38, 2556–2568

Leclerc, S., Smistad, E., Pedrosa, J., Østvik, A., Cervenansky, F., Espinosa, F., et al. (2019). Deep learning for segmentation using an open large-scale dataset in 2d echocardiography. *IEEE transactions on medical imaging* 38, 2198–2210

Li, M., Huang, K., Xu, Q., Yang, J., Zhang, Y., Ji, Z., et al. (2024). Octa-500: a retinal dataset for optical coherence tomography angiography study. *Medical image analysis* 93, 103092

Litjens, G., Toth, R., Van De Ven, W., Hoeks, C., Kerkstra, S., Van Ginneken, B., et al. (2014). Evaluation of prostate segmentation algorithms for mri: the promise12 challenge. *Medical image analysis* 18, 359–373

Luo, X., Liao, W., Xiao, J., Chen, J., Song, T., Zhang, X., et al. (2022). Word: A large scale dataset, benchmark and clinical applicable study for abdominal organ segmentation from ct image. *Medical Image Analysis* 82, 102642

Ma, J., Zhang, Y., Gu, S., Ge, C., Ma, S., Young, A., et al. (2024). Unleashing the strengths of unlabelled data in deep learning-assisted pan-cancer abdominal organ quantification: the flare22 challenge. *The Lancet Digital Health* 6, e815–e826. doi:https://doi.org/10.1016/S2589-7500(24)00154-7

Mazurowski, M. A., Clark, K., Czarnek, N. M., Shamsesfandabadi, P., Peters, K. B., and Saha, A. (2017). Radiogenomics of lower-grade glioma: algorithmically-assessed tumor shape is associated with tumor genomic subtypes and patient outcomes in a multi-institutional study with the cancer genome atlas data. *Journal of neuro-oncology* 133, 27–35

Porwal, P., Pachade, S., Kamble, R., Kokare, M., Deshmukh, G., Sahasrabuddhe, V., et al. (2018). Indian diabetic retinopathy image dataset (idrid): a database for diabetic retinopathy screening research. *Data* 3, 25

Setio, A. A. A., Traverso, A., De Bel, T., Berens, M. S., Van Den Bogaard, C., Cerello, P., et al. (2017). Validation, comparison, and combination of algorithms for automatic detection of pulmonary nodules in computed tomography images: the luna16 challenge. *Medical image analysis* 42, 1–13

Simpson, A. L., Antonelli, M., Bakas, S., Bilello, M., Farahani, K., Van Ginneken, B., et al. (2019). A large annotated medical image dataset for the development and evaluation of segmentation algorithms. *arXiv preprint arXiv:1902.09063*

Staal, J., Abràmoff, M. D., Niemeijer, M., Viergever, M. A., and Van Ginneken, B. (2004). Ridge-based vessel segmentation in color images of the retina. *IEEE transactions on medical imaging* 23, 501–509

[Dataset] Wasserthal, J. (2024). Spine segmentation from ct scans. doi:10.34740/KAGGLE/DSV/8422484

Zhang, Y., Xian, M., Cheng, H.-D., Shareef, B., Ding, J., Xu, F., et al. (2022). Busis: a benchmark for breast ultrasound image segmentation. In *Healthcare* (MDPI), vol. 10, 729

Zheng, X., Wang, Y., Wang, G., and Liu, J. (2018). Fast and robust segmentation of white blood cell images by self-supervised learning. *Micron* 107, 55–71
